# Human Colonization and Infection by *Staphylococcus pseudintermedius*: An Emerging and Underestimated Zoonotic Pathogen

**DOI:** 10.3390/microorganisms11030581

**Published:** 2023-02-25

**Authors:** Ikechukwu Benjamin Moses, Fernanda Fernandes Santos, Ana Cristina Gales

**Affiliations:** 1 Department of Internal Medicine, Division of Infectious Diseases, Escola Paulista de Medicina/Universidade Federal de São Paulo, Universidade Federal de São Paulo–UNIFESP, São Paulo 04039-032, Brazil; 2 Department of Applied Microbiology, Faculty of Sciences, Ebonyi State University, Abakaliki PMB 053, Nigeria

**Keywords:** staphylococci, zoonotic transmission, human infections, multidrug resistance

## Abstract

*S*. *pseudintermedius* is a known resident of the skin and mucous membranes and a constituent of the normal microbiota of dogs. It has also been recognized as an opportunistic and zoonotic pathogen that is able to colonize humans and cause severe diseases, especially in immunocompromised hosts. Most importantly, methicillin-resistant *S*. *pseudintermedius* (MRSP), which is intrinsically multidrug-resistant, has emerged with serious public health consequences. The epidemiological situation is further exacerbated with reports of its zoonotic transmission and human infections which have been mostly attributed to the increasing frequency of dog ownership and close contact between dogs and humans. Evidence on the zoonotic transmission of MRSP from pet dogs to humans (such as dog owners, small-animal veterinarians, and other people in close proximity to dogs) is limited, especially due to the misidentification of *S*. *pseudintermedius* as *S*. *aureus*. Despite this fact, reports on the increasing emergence and spread of MRSP in humans have been increasing steadily over the years since its first documented report in 2006 in Belgium. The emergence of MRSP strains has further compromised treatment outcomes in both veterinary and human medicine as these strains are resistant to beta-lactam antimicrobials usually prescribed as first line treatment. Frustratingly, the limited awareness and surveillance of the zoonotic transmission of *S*. *pseudintermedius* have underestimated their extent of transmission, prevalence, epidemiology, and public health significance. In order to fill this gap of information, this review focused on detailed reports on zoonotic transmission, human colonization, and infections by *S*. *pseudintermedius*, their pathogenic features, antimicrobial resistance profiles, epidemiology, risk factors, and treatment. In writing this review, we searched Web of Science, PubMed, and SCOPUS databases using the keyword “*Staphylococcus pseudintermedius* AND humans”. A phylogenetic tree to determine the genetic relatedness/diversity of publicly available genomes of *S*. *pseudintermedius* was also constructed.

## 1. Introduction

Although, nasal carriage of *Staphylococcus intermedius* (almost certainly *S. pseudintermedius* prior to its reassignment) was reported among humans who had contacts with dogs [1], *Staphylococcus pseudintermedius* was first described as a novel species of the *Staphylococcus intermedius* group (SIG) in 2005 [2]. Three other coagulase-positive staphylococci, *S*. *delphini*, *S*. *intermedius*, *S*. *cornubiensis*, and one coagulase-negative strain, *S*. *ursi*, also form members of the SIG [3]. *S*. *pseudintermedius*, and especially multidrug-resistant (MDR) strains, have been recognized to be causative agents of skin infections such as canine pyoderma and surgical wound infections, especially in dogs [4,5,6]. *S*. *pseudintermedius* has also been isolated in cats and horses [4] but is not as ubiquitous as reported in dogs, where up to 77–90% of healthy dogs are colonized [5,7]. Besides being a normal commensal of dogs, *S*. *pseudintermedius* is also known for its challenging opportunistic potential, especially in immunocompromised hosts. In addition, *S*. *pseudintermedius* has been implicated in various cases of human colonization and infections, mostly due to close contact between companion dogs and humans [8,9,10,11,12,13]. *S*. *pseudintermedius* started gaining attention in recent years with the emergence of methicillin-resistant *S*. *pseudintermedius* (MRSP), which is intrinsically resistant to beta-lactam derivatives and other non-beta-lactam antimicrobials. Considerable publications on antibiotic-resistant *S*. *pseudintermedius* mostly focus on isolates recovered from dogs, while few reports on human MRSP exist in the literature [14]. *S*. *pseudintermedius* colonization is very similar to *S*. *aureus* colonization in humans, with the human nares being the most common source of colonization in contrast to the pharynx and rectum in companion animals [5,6]. Moses et al. reported that the perineum of dogs was more colonized than their nares and mouth [8]. Even though substantial numbers of articles on *S*. *pseudintermedius* pathogenesis in companion animals have been published, its role in human infections is still largely understudied and underestimated. In most series of case reports, *S*. *pseudintermedius* has been reported to be associated with skin and soft tissue infections (SSTIs) and sometimes with invasive infections in humans (especially in immunocompromised dog owners) [6]. *S*. *pseudintermedius* has emerged as a very important zoonotic pathogen due to the similarities of its pathogenic arsenals when compared to that of *S*. *aureus* [7]. The zoonotic transmission of *S*. *pseudintermedius*, including the multidrug-resistant traits (such as MRSP) from companion animals to human guardians or other people in close and constant contacts with their pets, is still largely under-recognized and highlights a significant gap in understanding its epidemiology and pathogenesis in humans. *S*. *pseudintermedius* has also been reported as a common etiologic agent of urinary tract infections and a common cause of opportunistic infections when the host normal defenses are compromised [15,16]. The first report of *S*. *pseudintermedius* infection in a human was a case of cardiac device pocket infection in 2006 when it was initially misidentified as *S*. *aureus* [17]. Series of case reports which initially reported *S*. *intermedius* and *S*. *aureus* infections in humans have also been reclassified as *S*. *pseudintermedius* with the aid of more sophisticated identification techniques, especially matrix-assisted laser/desorption ionization–time-of-flight mass spectrometry (MALDI-TOF MS), species-specific PCR (*nuc* gene targeting), multilocus sequence typing (MLST), and whole-genome sequencing (WGS). This has further encouraged the reporting of *S*. *pseudintermedius* in human infections with a possibility of understanding its pathogenic potentials, epidemiology, and adaptations and estimating its prevalence in humans. Based on series of available new evidence, *S*. *pseudintermedius* has been recognized as one of the three most clinically important pathogens in the EU by the European Food Safety Authority [18]. Although the development and application of advanced microbiological technologies have really helped in the emerging literature on human *S*. *pseudintermedius* epidemiology, a lot remains to be studied. In this review, we highlighted detailed reports on the evidence of *S*. *pseudintermedius* colonization and transmission to humans and its pathogenic potentials and treatment, adaptations, risk factors, and epidemiology.

We downloaded articles by searching PubMed, Web of Science, and SCOPUS databases using the keyword “*Staphylococcus pseudintermedius* AND humans” in writing this review. Our search was from the year 2006, when *S*. *pseudintermedius* was first reported in a human, to 1st of December 2022. Our search strategy focused only on publications that reported *S. pseudintermedius* colonization/infection in humans. In our literature search, we excluded other publications that reported only animal colonization/infection by *S. pseudintermedius*. We also excluded articles that reported *S*. *intermedius*. This search resulted in the retrieval of 106 publications, out of which 97 publications on human colonization/infection by *S*. *pseudintermedius* and its zoonotic transmission were selected after the exclusion of duplicated articles, non-pertinent publications, and other publications that reported *S. intermedius*. We also evaluated the genetic relationship of *S. pseudintermedius* by constructing a phylogenetic tree on the Bacterial and Viral Bioinformatics Resource Center (BV-BRC) platform, using the codon tree method. The alignment of 100 single-copy coding genes was performed by applying the maximum-likelihood (RAxML) algorithm. Among 582 public genomes of *S. pseudintermedius*, a total of 147 were selected to construct a phylogenetic tree by using the following filters: “host common name”, “MLST”, and “genome quality = good”. Reference genomes, *S. pseudintermedius* SP_11304_3A and *S. aureus* NCTC 8325, were also included as a representative strain or outgroup, respectively. The GenBank accession numbers and the additional metadata are shown in Supplementary Table S1.

## 2. Identification of *S*. *pseudintermedius* at the Species Level

**Figure 1:**
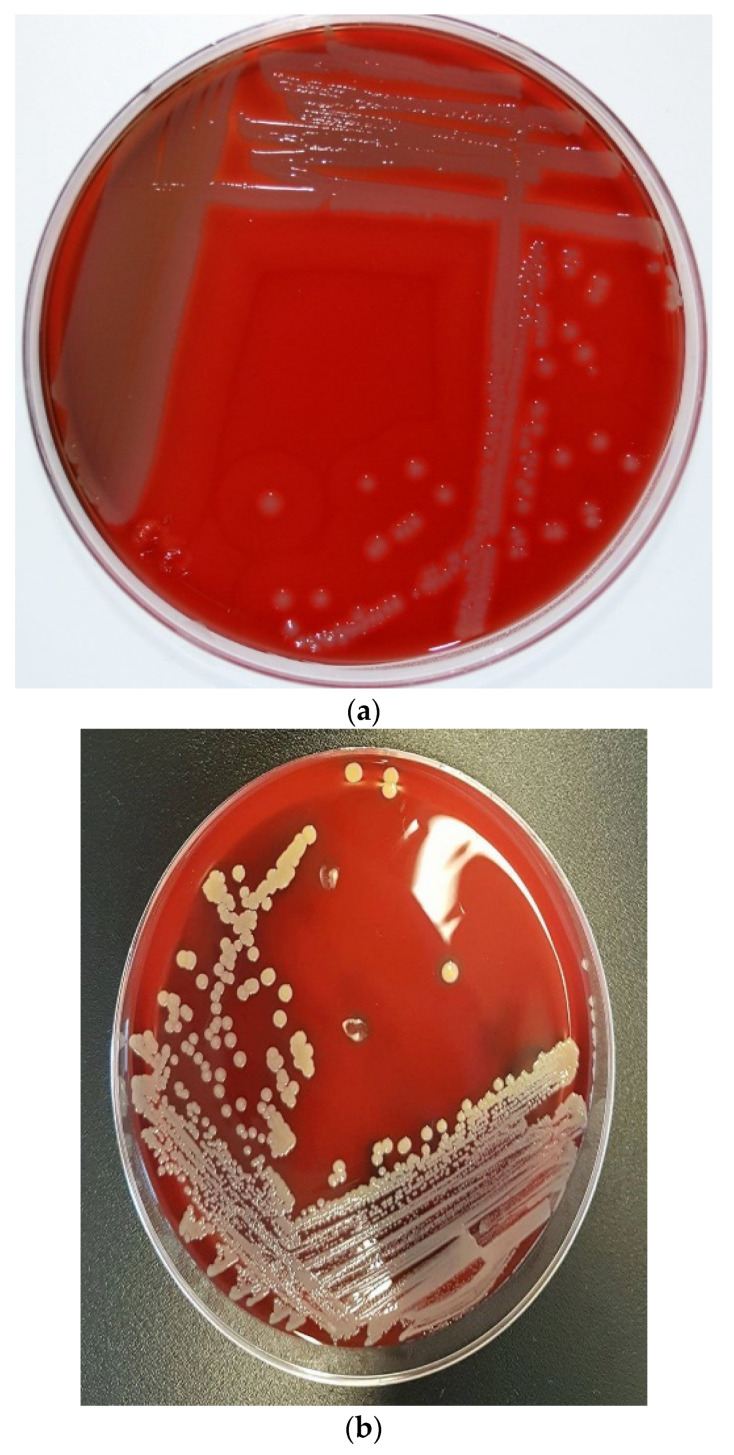

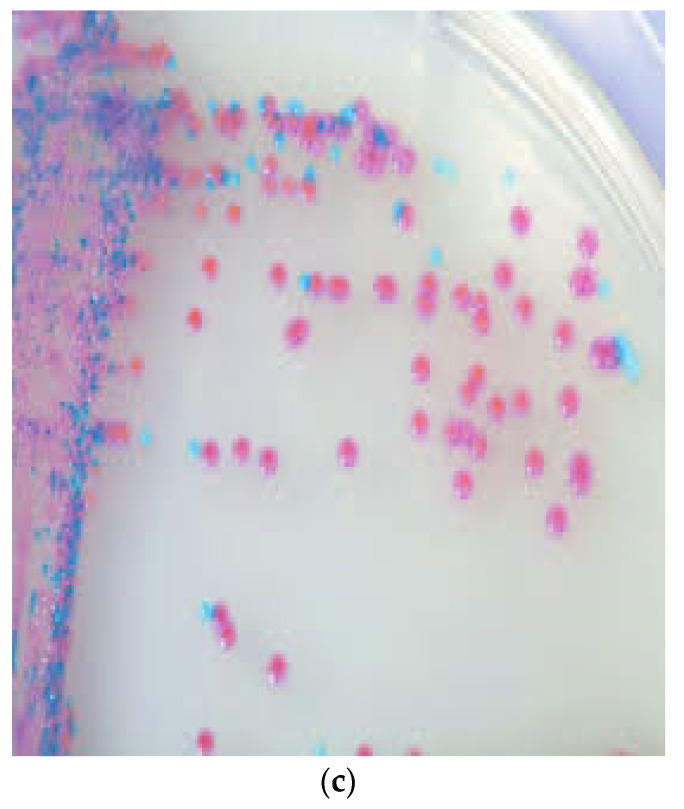
Colony morphology of *S*. *pseudintermedius* and its differentiation from *S*. *aureus*. (a) Creamy grey-to-white colonies of *S*. *pseudintermedius* on Columbia sheep blood agar. (**b**) *S*. *aureus* growth on Columbia sheep blood agar. (**c**) Mixed culture of Staphylococci on CHROMagar Staph aureus^TM^ (Oxoid, UK). Tiny blue colonies are *S*. *pseudintermedius*, while pink to mauve colonies are *S*. *aureus*.

Classical culture-based techniques have been the mainstay of clinical microbiology in previous centuries before the introduction of automated identification systems. The introduction of the API test kits and its automated versions (such as VITEK systems) influenced some level of success in microbial pathogen identification [19]. However, the database of these automated systems is well-populated by bacterial species commonly reported in human microbiology and have less representation of some emerging zoonotic pathogens, such as *S*. *pseudintermedius*. This negatively affected the identification of *S*. *pseudintermedius* since it is biochemically and phenotypically difficult to differentiate from other staphylococci (e.g., *S. intermedius*) [5,6,8]. In some automated systems, *S*. *pseudintermedius* is usually misidentified as *S*. *aureus* [4]. This has contributed greatly to the underestimation of the actual prevalence of *S. pseudintermedius* in both veterinary and human medicine. In some automated systems, *S. pseudintermedius* is usually misidentified as *S. aureus* [3]; however, there has been good improvement in the accurate identification of *S*. *pseudintermedius* as most automated systems are now updated with new software. *S*. *pseudintermedius* grows as small blue colonies on CHROMagar Staph aureus^TM^ and as small, creamy grey-to-white, round 1–3 mm colonies with β-haemolysis on Columbia sheep blood agar [20]. The morphological appearances of *S*. *pseudintermedius* and its differentiation from *S*. *aureus* are shown in Figure 1. They are Gram-positive cocci in bunches with positive catalase test, typical of other staphylococci [20]. In addition, *S*. *pseudintermedius* is tube coagulase-positive but may be misidentified as coagulase-negative with the slide coagulase and commercial latex agglutination tests due to its slow and poor response [5,21]. The similarities in morphological and biochemical test results of *S*. *pseudintermedius* and *S*. *aureus* have led to series of misdiagnosis in human diagnostic laboratories, especially in most low- and middle-income countries without the financial capability to afford highly advanced identification machinery/techniques. However, despite this economic challenge, the morphology on agar plates and some biochemical tests such as acetoin production, hyaluronidase tests, mannitol fermentation, pyrrolidonyl arylamidase, polymyxin B susceptibility, beta-galactosidase production, and carbohydrate fermentation tests such as mannitol, maltose, and trehalose have proven to be useful in identifying and differentiating *S*. *pseudintermedius* from *S*. *aureus* [5,20,22] (Table 1). The introduction of new advanced technologies such as MALDI-TOF MS, PCR amplification of species-specific target genes such as the thermonuclease (*nuc*) gene [23], which differentiates *S*. *pseudintermedius* from *S*. *aureus*, and other SIG group members, and DNA sequencing techniques (such as multilocus sequence typing and whole-genome sequencing) in recent years has strongly helped in overcoming the disadvantages of the classical biochemical identification techniques. Multilocus sequence typing (MLST) is an important DNA sequencing technique for determining the epidemiological identities of pathogens [24]. In 2007, the first MLST scheme designed to evaluate the population genetic structure of the *Staphylococcus intermedius* group (SIG), which includes *S*. *pseudintermedius*, *S*. *intermedius*, and *S*, *delphini*, was based on 5 gene loci [25]. This scheme detected two major MRSP sequence types (STs): ST71 in Europe and ST68 in North America [6]. In 2013, Solyman et al. [26] launched the first species-specific MLST scheme, which is publicly available in a database (http://pubmlst.org/spseudintermedius/, accessed on 1 December 2022). As of December 2022, a total of 582 *S*. *pseudintermedius* genomes (mostly from dog) have been deposited on the MLST database. Whole-genome sequencing using an Oxford Nanopore MinION device and illumina sequencing platforms have proven to be very valuable in deciphering the complete genetic properties, epidemiological identities, and pathogenic potentials of *S*. *pseudintermedius* [4,6,7,25,26].

## 3. *S. pseudintermedius* Resistance to Beta-Lactams and Emergence of MRSP

In the last decade, the antimicrobial resistance patterns of *S*. *pseudintermedius* isolated from humans have not been well studied due to the inability of phenotypic and automated methods to properly identify and differentiate the pathogen from other human pathogens, such as *S*. *aureus* [27]. It is therefore particularly challenging and difficult to make definite and comprehensive statements on the resistance patterns of human *S. pseudintermedius*. However, despite these challenges, the emergence of antibiotic-resistant *S*. *pseudintermedius* with great relevance to human medicine has been noted from previous reports. Over the years, *S*. *pseudintermedius* has been observed to be frequently resistant to penicillinase-susceptible penicillins such as amoxicillin, ampicillin, and penicillin G. Of note, MRSP are considered to be resistant to beta-lactams according to the Clinical Laboratory Standards Institute (CLSI) and European Committee on Antimicrobial Susceptibility Testing (EUCAST) recommendations based on series of evaluated reports [14,28]. Oxacillin has been reported to be a better predictor of MRSP when compared to cefoxitin, which is mostly used for the accurate prediction of methicillin resistance in *S*. *aureus* and other coagulase-negative staphylococci [14,27,28]. To properly identify MRSP isolates, Wu et al. [28] carried out a study which evaluated cefoxitin and oxacillin disks, together with their MIC results, using 115 SIG isolated from veterinary and human clinical samples. They reported that oxacillin was a better predictor of MRSP isolates as it more accurately detected *mec*A resistance genes compared with cefoxitin [28]. Due to this and other series of reports, specific oxacillin breakpoints for MRSP detection with high reliability for *mec*A detection is oxacillin MIC of ≥0.5 mg/L (broth and agar dilution) and an inhibition zone diameter (IZD) of ≤17 mm (disc diffusion) according to the CLSI recommendations for isolates from both humans and animals [29]. In contrast to CLSI, EUCAST only defined an IZD of < 20 mm for MRSP detection without an available MIC breakpoint for agar and broth dilution [30]. The immunochromatographic detection of an altered penicillin-binding protein (PBP2a) of MRSP in contrast to its serological detection has also proven to be reliable in identifying methicillin resistance [6]. However, with recent advancement in molecular techniques, and also for high reliability of results, the phenotypically identified methicillin-resistant isolates must have a *mec*A gene to be genetically classified as MRSP [31]. Increasing emergence of MRSP in companion animals, especially among dogs and cats, has been discussed in other publications and reviews [32]; however, its emergence in human medicine, including its alarming rates of occurrence have not been properly addressed. The resistance of MRSP isolates to penicillinase-stable penicillins and oxacillin has attracted more attention in both veterinary and human medicine. In previous microarray studies in North America and Europe, 99% (102/103) MRSP isolates were identified to possess the *bla*Z gene which codes for a narrow-spectrum beta-lactamase [7,33]. Besides harboring the *bla*Z gene, MRSP are also known to possess the staphylococci cassette chromosome *mec* (SCC*mec*) gene, a mobile genetic element which harbors the *mec*A gene that codes for an alternative binding protein, PBP2a. In addition, arrays of SCC*mec* elements have been observed among different genetic lineages of MRSP, thereby suggesting that *mec*A genes might have been acquired multiple times by different *S*. *pseudintermedius* [4,32]. The transfer and exchange of SCC*mec* elements among different staphylococcal species is of significant public health concern as the staphylococci strains could evolve into superbugs causing “difficult-to-treat” infections. Series of reports tracing the origin of MRSP-specific SCC*mec* elements suggest that they might have been derived from or associated with *S. aureus* as well as other coagulase-positive staphylococci strains [34,35]. In humans, MRSP is rarely reported; however, the transfer potential of SCC*mec* elements and other resistance genes from MRSP to other staphylococcal species such as *S*. *aureus* has been hypothesized and is likely possible. It has been reported that the type of SCC*mec* elements present in MRSP seem to differ from those harbored by MRSA from genome sequencing results [36,37]. The SCC*mec* II-III hybrid, which is made up parts of previously identified SCC*mec* types II and III of MRSA, has been reported in *S*. *pseudintermedius* [36,38]. A total of 72.8% (75/103) MRSP isolates recovered from canines in North America, Europe, and Asia were reported to harbor the SCC*mec* II–III hybrid, while the remaining isolates harbored either SCC*mec* types III, IV, V, VII, or were not typeable [39]. Interestingly, most MRSP isolates recovered from human infections usually harbor the SCCmec III gene. A case of an implanted port catheter system infection caused by MRSP ST71-SCC*mec* III in a dog owner patient with hepatocellular carcinoma was reported in Japan [38]. This MRSP ST71-SCC*mec* III strain is a well-known epidemiological clone mostly isolated from dogs in Asia. MRSP harboring the SCC*mec* II–III genes have also been reported in various human infections, especially among people in close contact with dogs [14,40,41,42]. MRSP isolates carrying SCC*mec*V genes have also been reported in human infections [43,44] but in low frequency when compared to SCC*mec* II–III hybrid. MRSP has been recognized as an important pathogen in veterinary medicine with similar propensity in comparison to the healthcare-associated methicillin-resistant *S*. *aureus* (MRSA) in human medicine [39,45]. In Europe, the first case of MRSP was published in 2007 [46]. Since then, the occurrence of MRSP has been increasing steadily in both veterinary and, more recently, human medicine as it has been fingered as the cause of most wound infections and device-associated case reports, especially among dog owners or individuals in close contact with infected dogs. MRSP clones reported in North America (ST68), Europe (ST71), and Asia (ST45) have been observed to exhibit MDR to oral and most parental antimicrobials approved for veterinary use [4], thus creating a new and increasing pressure to use antimicrobials reserved for human medicine. This is worrying and calls for more surveillance programs to properly monitor the zoonotic transmission and pathogenesis of MRSP in humans.

## 4. Virulence Factors of *S*. *pseudintermedius*

*S*. *pseudintermedius* is equipped with a variety of virulence factors such as enterotoxins, coagulase, protease, haemolysin, *siet* exfoliative toxin, and *sec_canine_* enterotoxin, which empowers it to overcome the immune system arsenals and further increase the severity of infections in the infected host [20,21]. Of great interest is the possession of a leukotoxin virulence gene known as *luk* by *S*. *pseudintermedius*, which is similar to the Panton–Valentine leucocidin (*PVL*) found in *S*. *aureus* [20,22]. The SpsQ and protein A found in *S*. *pseudintermedius* are analogous to those found in *S*. *aureus* [47,48]. SpsD and spsL surface proteins, which facilitate adherence to human fibronectin, fibrinogen, and cytokeratin, have also been reported [49]. Some putative virulence features involved at various pathogenic stages of *S*. *pseudintermedius* infections (adhesion, immune evasion, and spread) have been described [50]. Most of the *S*. *pseudintermedius* virulence factors are similar to the ones described in *S*. *aureus*, thus explaining the similarity in severity of infections caused by both bacterial pathogens.

### Implications of S. pseudintermedius Virulence Factors in Human Health

*S*. *pseudintermedius* infections in humans have been reported, but the roles of their virulence factors in human infections is largely understudied and therefore underestimated. Although human *S*. *pseudintermedius* infections are scarce because of reported low carriage frequency when compared to *S*. *aureus*, interestingly, an in vitro study showed that *S*. *pseudintermedius* expressed similar pathogenic potentials to *S*. *aureus* towards human cells [51].

Low *S*. *pseudintermedius* carriage frequencies even among high-risk groups such as pet guardians/owners and small-animal veterinarians, including other people in close contact with pets, have been reported in some studies. This might not be a definitive representation of the actual prevalence frequencies because misdiagnosis of *S*. *aureus* could be an important limiting factor underestimating its prevalence in humans. For example, a study in Sweden reported that 13 out of 101 isolates previously identified as *S*. *aureus* from human samples of infected dog-bite wounds were in fact found to be *S*. *pseudintermedius* after using molecular techniques. In addition, the isolates were found to harbor *Luk*F/S-I, *siet*, *se-int*, *exp*A, *exp*B, and *Sec*_canine_ genes [22]. A similar study that investigated the zoonotic transmission of *S*. *pseudintermedius* between dogs and dog guardians in different households revealed that three dog guardians (4.5%) were colonized by *S*. *pseudintermedius* [52]. Gomez-Sanz et al. [52] also observed that one of the *S*. *pseudintermedius* colonizing a dog guardian was similar to the one isolated from the corresponding dog. Interestingly, two *S*. *pseudintermedius* strains isolated from the dog guardians harbored the *exp*A gene (an exfoliative toxin gene), further confirming the hypothesis that companion animal ownership is a significant risk factor for the zoonotic transmission of pathogenic *S*. *pseudintermedius*, including the MDR and MRSP strains. *Luk*F/S-I and *siet* genes have also been reported in MRSP causing wound infections in a cluster of four cases in a tertiary hospital [42]. The possible animal source of the infections could not be identified as a follow-up sampling of the patients’ pets was not done. *S*. *pseudintermedius* harboring *siet* exfoliative genes and *Luk*F/S-I genes were also isolated from a dog and its guardian suffering from severe skin infection [53]. *S*. *pseudintermedius* has also been reported to adhere to human fibronectin with greater internalization ability, intracellular persistence, and cytotoxicity using human osteoblasts than *S*. *aureus* [54]. A biofilm-producing antibiotic-resistant *S*. *pseudntermedius* strain was isolated from the wound of a human patient with chronic lymphoblastic leukaemia who had close contact with a companion dog [55]. In addition, the biofilm produced by the *S*. *pseudintermedius* pathogen was resistant to many antibiotics, including the last-line antimicrobials such as linezolid, tigecycline, and vancomycin. In another study, *S*. *pseudintermedius* strains isolated from humans were reported to harbor *sea*, *seb*, *sec*, *see*, and *tst*1 virulence genes [56]. The real significance of findings reporting the zoonotic transmission of *S*. *pseudintermedius* with their virulence factors from companion animals to their human guardians is difficult to evaluate because of the low number of isolates included in most of these studies.

## 5. The Zoonotic Transmission, Colonization/Infections, and Epidemiology of *S*. *pseudintermedius* in Humans

The close contact between companion animals (especially dogs and cats), their human guardians/owners, and other people such as small-animal veterinarians increases the likelihood of *S*. *pseudintermedius* (a normal commensal of dogs) adaptation in humans, as already mentioned. Veterinarians (3.9%, 5/128) have been reported to be colonized by MRSP [14]. A cohort study reported that human patients with a median age of 61 and older may be at higher risk of being infected by *S*. *pseudintermedius* from their dogs. Phumthanakorn and Prapasarakul [57] performed assays to determine the adherence ability of five MRSP isolates belonging to different sequence types (ST45, ST433, and ST733) from different sources on human corneocytes and reported that three isolates of MRSP ST45, the major clone in Thailand, had the strongest ability to adhere to human corneocytes among all the tested STs. This further highlights the epidemiological success of ST45 in Asia when compared to ST71, a previously successful clone in Europe. Interestingly, both ST45 and ST71 have disseminated globally, with ST71 being the most reported in human infections globally. In another study, Latronico et al. [58] compared the in vitro adherence potentials of four MRSP strains belonging to ST71, two non-ST71 strains, and three genetically unrelated MSSP on human corneocytes. They observed that MRSP ST71 strains showed greater adherence than MRSP non-ST71 and MSSP. This further proves the epidemiological success of human colonization by MRSP ST71 and its increasing global dissemination in series of case reports, especially among immunocompromised individuals who have contacts with dogs.

As noted earlier in this review, studies describing the zoonotic transmission, colonization, and infection of humans by *S*. *pseudintermedius* are few due to its misidentification as *S*. *aureus* and also due to the inability of most diagnostic laboratories to afford the use of newer technologies such as MALDI-TOF MS, species-specific gene targeting by PCR, MLST, and whole-genome sequencing, especially in developing countries. As a result of this, it is somewhat challenging to estimate its actual frequency of zoonotic transmission, human colonization and infection, and its current epidemiology. However, despite these challenges, we were able to give detailed information on most reports of zoonotic transmission, human colonization and infection, and the epidemiology of *S*. *pseudintermedius* in humans from 2006 when *S*. *pseudintermedius* was first reported in a human infection to 1 December 2022 [4,6,9,10,11,12,13,14,15,16,17,20,22,38,40,41,42,43,44,52,53,55,59,60,61,62,63,64,65,66,67,68,69,70,71,72,73,74,75,76,77,78,79,80,81,82,83]. A summary of all reported human *S*. *pseudintermedius* infections, zoonotic transmission, and colonization from 2006–1st of December 2022 is shown in Table 2. Most of the literature on human infections due to *S*. *pseudintermedius* cited case reports due to skin and soft tissue infections (SSTIs), device-associated, and invasive infections, especially among hospitalized patients who have or had prior contact with companion animals. In a case series, 12.9% (13/101) of *S*. *pseudintermedius* isolates previously identified as *S*. *aureus* were associated with dog-bite wounds of patients. In one of the largest case series involving 24 patients (18 SSTIs, 1 prosthetic joint infection, 1 skin infection, 1 lung infection, 1 bloodstream infection, 2 invasive infections–a monomicrobial prosthetic joint infection, and a fistula-associated bloodstream infection), *S*. *pseudintermedius* was identified as a significant component of a polymicrobial infection in 91.7% of the observed cases [6]. This study reported that 95.4% of the observed patients were dog owners and had close contact with their dogs before the *S*. *pseudintermedius* infections. Most of the infections reported in this case series were mild to moderate, so patients were primarily treated with oral antibiotics and managed as outpatients [6].

MRSP is known to originate from dog reservoir in contrast to MRSA strains whose main reservoir is human. MRSP is the primary staphylococcal species responsible for a plethora of dog infections [4]; however, its zoonotic transmission to humans is of public health significance even though *S. aureus* is currently a greater concern in human medicine. One (7.7%) MRSP isolate was reported among 13 *S*. *pseudintermedius* isolates recovered from dog bite wound infections in a case series [22]. Somayaji et al. [6] also isolated MRSP isolates which belonged to sequence types (ST71 and ST181) mostly reported in Europe in 3 patients [6,84]. ST71 and ST181 have been noted to also possess multidrug resistant traits, but so far, no MRSP resistance to vancomycin, daptomycin, or linezolid has been reported in isolates from humans. MRSP ST71 and ST 68 have been reported to be predominant in Europe and North America, respectively [7]. Previously, the MRSP Sequence Type 71 carrying the SCC*mec* III (MRSP ST71-SCC*mec* III) was regarded as an epidemic European clone usually isolated from dogs, but today, it is highly disseminated worldwide and recognized to be the major MRSP clone infecting humans in close contact with dogs, especially among the immunocompromised such as individuals with open wounds and medical devices [6,9,38,40,42]. Other *S*. *pseudintermedius* STs such as ST45, ST241, ST1337, ST1412, ST155, ST673, ST686, ST181, ST158, and ST233 have also been reported in human colonization and infections [22,41,44,64,70,73,81,85]. A study reported 5.6% *S*. *pseudintermedius* carriage rate among dog owners in a household [52,86]. Within households, *S*. *pseudintermedius* may also be transmitted through the fecal-oral routes due to the tendency of *S*. *pseudintermedius* to colonize the rectum and pharynx of dogs [5]. The zoonotic transmission of *S*. *pseudintermedius* in nosocomial settings may also occur [42].

## 6. Evolutionary Relationships of *S*. *pseudintermedius* Isolated from Humans and Animals: An Assessment of Their Genetic Relatedness/Diversity

To understand the genomic relationship among *S. pseudintermedius* isolated from humans and animals, a phylogenetic tree was built using the genomes deposited in the public database from 1999 to 2019. Publicly available genomes of *S*. *pseudintermedius* isolated from humans are few when compared to the ones from companion animals. To date, only 356 out of the publicly available 582 *S*. *pseudintermedius* genomes have their host information associated with them on the database. Most of the *S. pseudintermedius* strains were recovered from dogs (*n* = 328), followed by humans (*n* = 17), horses (5), cats (4), cows (*n* = 1), and seals (*n* = 1).

**Figure 2:**
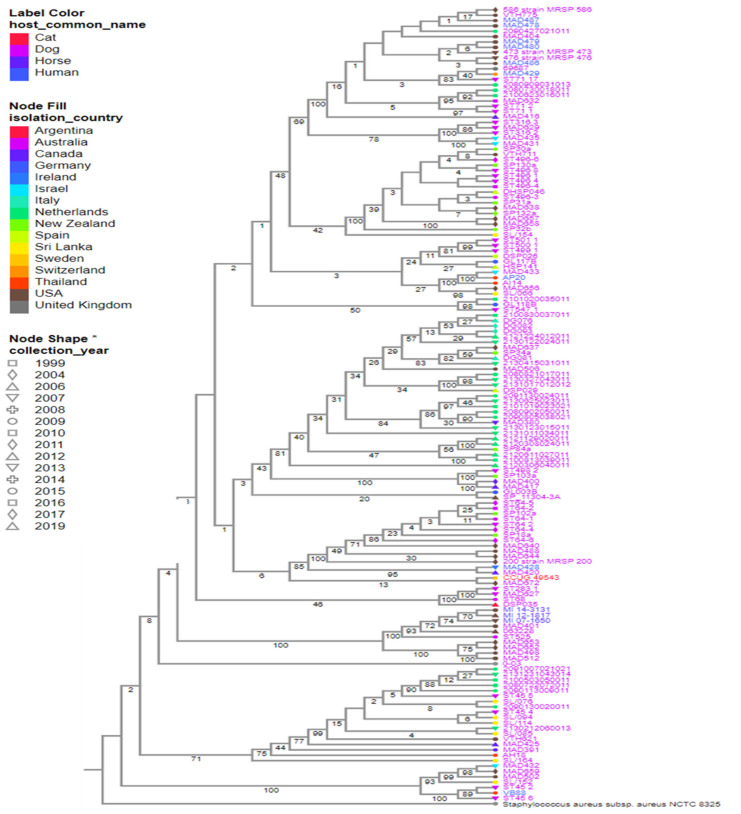
Strains of 147 *S. pseudintermedius* isolated from humans and animals worldwide. Reference genomes, *S. pseudintermedius* SP_11304_3A and *S. aureus* NCTC 8325, were also included. The color of the strain’s name represents the hosts, the color of the nodes indicates the country of isolation, and the node shape indicates the year of isolation. Estimated confidence is shown on each internal branch of the tree. * is just a form of emphasis on Node shape based on years. It was not used to represent the collection year type.

**Figure 3:**
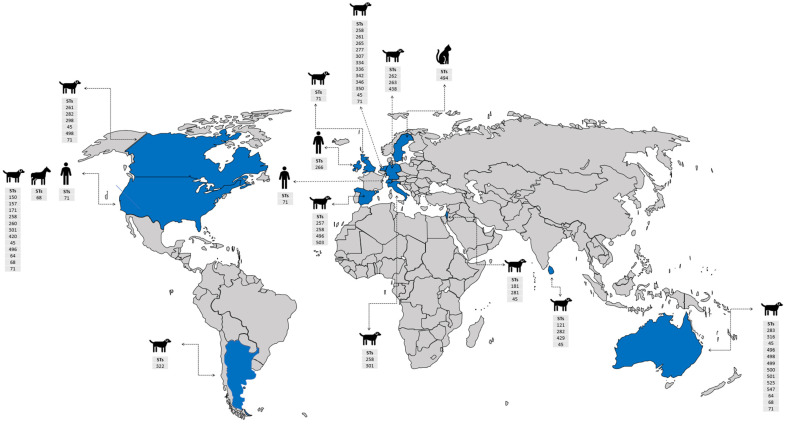
World map showing countries with *S*. *pseudintermedius* reports, their MLSTs, and hosts. The information included in the map was based on selected criteria of the phylogenetic tree. Countries with *S*. *pseudintermedius* reports are shown in blue. 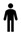: human, 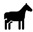: horse, 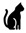: cat, and 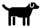: dog.

Herein, a total of 147 representative genomes of *S. pseudintermedius* isolated worldwide were selected to create the phylogeny of this species, using the following filters: “host common name”, “MLST”, and “genome quality = good”. Based on our analysis, the strains isolated from humans (MAD-487, -478, -479, -480, -486) and dogs (MRSP-586, -473, -476, VTH-775, MAD-404) in the USA in different years were closely related (Figure 2). A similar relationship was observed between strains AP20 and AI14 isolated from a human and a dog in Thailand, respectively (Figure 2 and Figure 3). In addition, strains MI-143131, -12-1817, and -07-1650 isolated from horses and MAD-401 and 063228 isolated from dogs were also genetically related (Figure 2). These results indicated a likely zoonotic and anthropozoonotic transmission of these *S*. *pseudintermedius* strains between humans and animals in the studied regions. The phylogenetic tree also showed that some of the related clones were recovered from different countries and different hosts, such as the strain CCUG-49543 from a cat in Sri Lanka and MAD-672 from a dog in the USA; VB88 from a human in Argentina and ST452 from a dog in Australia; thus, suggesting a close ancestry between them (Figure 2 and Figure 3).

Based on the publicly deposited genomes, the most frequent ST observed among the strains was ST45 (*n* = 22), followed by ST71 (*n* = 21), ST496 (*n* = 16), ST258 (12), ST64 (*n* = 10), and ST68 (*n* = 6) being the least (Supplementary Table S1). The other STs observed had four or fewer representative strains (Supplementary Table S1). Interestingly, the closest clones recovered from the same country share identical STs, such as MRSP-473, -586, -476, MAD-404, -478, -480, -487, -479, -486, and VTH775 belonging to the ST71; MAD401, 063228, MI-12-1817, -07-1650, and -14-3131 belonging to ST68; and AP20 and AI14 belonging to ST181. Additionally, the strains recovered from different countries such as VB88 and ST452 belonged to the same ST (45). Although the publicly available genomes of *S. pseudintermedius* do not completely give an actual representation of the circulating STs, results of the phylogenetic tree analysis of the public genomes showed a worldwide epidemiological dissemination of ST45 and ST71 as the most dominant *S*. *pseudintermedius* STs in animals (especially dogs) and humans. This further highlights their lack of host specificity and the possibility of interspecies transmission.

## 7. Factors Associated with Acquisition of MRSP

Although the primary variables contributing to the rising frequency of MRSP have not yet been definitively discovered through studies, various potential risk factors have been noticed throughout time [87,88]. Of note, veterinary clinics and hospitals play important roles in the spread and zoonotic transmission of MRSP. Even though there are low numbers of reports on colonization, MRSP is even gradually being recognized as an occupational risk for veterinarians [14,40,41,52,62,86,89,90,91]. A carriage rate of 4% was reported among small-animal dermatologists [14]. Over the years, there has been an interesting significant increase in the number of case reports on MRSP infections in humans who have close contact with dogs [9,63,92]. Risk populations and recognized factors associated with the spread and transmission of MRSP infections include dog owners from MRSP-positive households [93], veterinarians [94], exposure to medical hospitals and environments, extensive wounds [93,94], underlying diseases, severity of illness, advanced age, gastrointestinal surgery, transplantation, prolonged hospitalization [94], exposure to invasive devices of all types (especially central venous catheters), immunosuppression [94,95], and antimicrobial administration to a dog or an owner [4,95,96,97]. It has been reported that people with very close contact with infected animals have a higher risk of being MRSP-positive [4]. This further supports that MRSP could be a more common bacterial pathogen in humans than previously estimated [22].

## 8. Antimicrobial Choices for the Treatment MRSP Infections and Its Challenges

The emergence of MRSP presents a new challenging public health problem to both veterinary and human medicine because therapeutics options are limited [82]. Due to the unpredictability of MRSP susceptibility to antimicrobials (especially non-beta-lactams), it is imperative to always conduct antimicrobial susceptibility testing in the treatment of MRSP infections in order to choose the best and most effective antimicrobial which will help to curtail the increasing spread of these zoonotic pathogens. Interestingly, aminoglycosides, tetracyclines, chloramphenicol, and rifampicins (also known as rifampins in some countries) are antibiotics to consider in the treatment of MRSP infections if antimicrobial susceptibility results indicate good activity. In contrast to the community-acquired MRSA in human medicine, MRSP originating from veterinary and human medicine have been noted to usually exhibit resistance to non-beta-lactam drugs such as trimethoprim–sulfamethoxazole, fluoroquinolones, lincosamides, macrolides, and tetracyclines [7,31,82].

## 9. Conclusions

*Staphylococcus pseudintermedius* is a well-recognized veterinary pathogen, mostly colonizing and causing diseases in dogs. However, in the last decade, pet (especially dog and cat) ownership in modern society has significantly increased [98]. This bacterial species could be transmitted from dogs to humans (especially dog owners and small-animal veterinarians) because of their frequent close contact. In fact, human colonization and infections due to *S. pseudintermedius* have been increasingly reported.

Nevertheless, to properly understand the prevalence, pathogenesis, and epidemiology of *S*. *pseudintermedius* in human medicine, it is important to precisely identify all the CoPS that are frequently misdiagnosed as *S*. *aureus* in human clinical samples. In routine microbiological diagnosis, phenotypic test results could be combined with host information to better distinguish these *Staphylococcus* bacteria.

Of special interest is the emergence of MRSP, which is usually intrinsically resistant to beta-lactam derivatives and exhibits multidrug resistance traits to other non-beta-lactam antimicrobials such as the sulfonamides, fluoroquinolones, lincosamides, macrolides, and tetracyclines. The emergence of MRSP represents a loss of antimicrobial effectiveness and further complicates the treatment of MRSP infections in both veterinary and human medicine. Furthermore, global epidemiological reports indicated that most MRSP strains infecting humans belong to ST45 (CC45), a dominant clone in Asia, and ST71 (CC71), a major epidemic clone found in dogs and also a previously known dominant clone in Europe, thus further reiterating its global epidemiological success. Finally, this review revealed that *S. pseudintermedius* strains should be evaluated from a One Health perspective, considering the close contact between humans and companion animals, and their consequent colonization/infection which will draw back the gains of One Health.

## 10. Future Outlook

The outlook to properly track and understand the zoonotic transmission of *S*. *pseudintermedius* will be for healthcare providers or physicians to consider asking patients infected with staphylococci routine questions on “contacts with animals”. Inadequate, incomplete, or lack of appropriate diagnosis is an important factor that has exacerbated the increasing antimicrobial resistance menace in both veterinary and human medicine due to antimicrobial misuse or abuse. The development, implementation, and application of advanced microbiology technologies with high discriminatory power and high efficiency have helped in the proper identification of *S*. *pseudintermedius* with a progressing opportunity in understanding and evaluating its pathogenesis, prevalence, epidemiology, and zoonotic transmission potentials. In the interim, the development or identification of important phenotypic tests which differentiate *S*. *pseudintermedius* from other members of staphylococci will be valuable in contributing to the correct estimation of *S*. *pseudintermedius* prevalence, especially for smaller diagnostic laboratories that cannot afford highly sophisticated identification techniques/machinery. The development of an ATLAS picture album, which would contain a series of morphological images of *S*. *pseudintermedius* and its differentiation from CoPS on different commercial or self-formulated culture media, will be very useful in screening for suspected *S*. *pseudintermedius* colonies before identification confirmation. Further prospective and longitudinal studies on human *S*. *pseudintermedius* will be very valuable to properly decipher and understand the transmission, risk factors, pathogenic potentials, and epidemiology of this opportunistic and zoonotic pathogen.

## Figures and Tables

**Table 1:** Phenotypic Identification of *S*. *pseudintermedius* and its differentiation from *S*. *aureus*.

Test	*S*. *pseudintermedius*	*S*. *aureus*
Catalase	+	+
Coagulase	+	+
DNAase	+	+
Haemolysis	β-haemolysis	Double zone haemolysis
Trehalose	+	+
Maltose	+	+
Clumping factor	-	+
Pigment	-	+
Pyrrolidonyl arylamidase (PYR)	+	-
β-galactosidase	+	-
Acetoin production	-	+
Mannitol fermentation	-	+
Polymyxin susceptibility	Susceptible	Resistant
Hyaluronidase	-	+

**Table 2:** Summary of reports on *S*. *pseudintermedius* colonization and infections in humans from 2006–2022.

Year	Country	Infection Type and Number of Cases	Author(s)
2006	Belgium	Infection of an implantable cardioverter-defibrillator (ICD) device in a 60-year-old male patient	[17]
2009	USA	Colonization of two dog owners by MRSP which originated from their dogs suffering from skin infections.	[43]
2009	Canada	Human colonization by *S*. *pseudintermedius* (SP)	[59]
2010	Taiwan	A case of catheter-related bacteremia due to SP in a 6-year-old boy with with hemophilia B after dog exposure	[10]
2010	Switzerland	Infection of an adult male with a history of recurrent rhinosinusitis by MRSP ST71	[9]
2011	France	A case of SP infection related to the device associated with endocarditis	[60]
2011	Netherlands	Colonization of dog owners and veterinarians by MRSP	[4]
2011	Netherlands	Human colonization by MRSP in a dog-owning household	[61]
2011	Hong Kong	Nasal colonization of a veterinary personnel by MRSP ST71	[40]
2011	Germany	Nasal colonization of dog owner by MRSP	[62]
2011	Italy	MRSP nasal carriage by small-animal dermatologists	[14]
2013	Spain	Nasal carriage of SP ST142 in pet-owning household members	[52]
2013	Italy	MRSP infection in a 65-year-old male bone marrow transplant recipient	[63]
2013	South Korea	Colonization of a healthy female veterinarian by MRSP ST233	[64]
2014	Sweden	Cluster of MRSP clone ST71-J-t02-II–III infections in 4 elderly patients with wound infections due to diabetes mellitus and recurrent venous ulcers in a tertiary hospital.	[42]
2014	Thailand	Colonization of veterinarians and dog owners by MRSP ST45, 68, and novel STs, including 169, 178, 181, and 183	[41]
2015	Sweden	MRSP infection in humans due to dog bite wounds	[22]
2015	Italy	Infection of a 65-year-old leukemic patient wound in the periumbilical region who underwent a bone marrow transplant by biofilm-producing MRSP	[55]
2015	Spain	Nasal carriage of SP in two non-infectious patients	[65]
2016	USA	First case series of rhinosinusitis SP infection in humans	[66]
2016	Canada	Human infections due to SP in 24 cases of SSTIs and invasive cases, including prosthetic joint, bloodstream, and lung infections	[6]
2017	UK	Severe skin infection caused by SP in a 47-year-old dog owner	[53]
2017	UK	Invasive spinal infection with SP associated with a 15-year-old spinal fixation device in a 60-year-old woman	[67]
2017	Spain	Human infections caused by SP ST241, 521, 719, 720 in a hospital	[68]
2018	Poland	Human colonization by SP	[69]
2018	Thailand	Colonization of dog owners by MRSP	[70]
2018	Germany	MRSP carriage among employees of a small-animal hospital	[71]
2018	Portugal	Nasal colonization of healthy humans by MRSP ST71-SCCmecII–III	[72]
2019	New Zealand	Nasal carriage of SP in 69 patients with granulomatosis with polyangiitis	[73]
2020	USA	First case of peritoneal dialysis-associated peritonitis caused by SP in a 39-year-old female patient	[74]
2020	Argentina	MRSP infection of surgical wound in an 86-year-old female patient with history of hypertension, deep vein thrombosis, and chronic ulcers after vene cava filter placement	[43]
2020	Japan	An implantable venous access port infection due to SP in a 41-year-old dog owner	[75]
2020	Canada	A case of bacteremia due to SP in a 4-month-old pediatric oncology patient	[11]
2020	Canada	Persistent SP infection in an adult female oncology patient including colonization of the tip of an indwelling catheter.	[11]
2021	USA	MRSP infection in case of a 50-year-old female with bilateral lung transplant	[12]
2021	Netherlands	Human colonization by MRSP	[76]
2021	Japan	Implanted port catheter system infection by MRSP ST71-SCC*mec* III in a dog owner patient with hepatocellular carcinoma	[38]
2021	USA	A case of septic arthritis due to SP in an otherwise healthy child	[77]
2021	USA	A case of postprocedural urosepsis in an elderly patient with recent bilateral ureteral stent placement due to SP	[15]
2021	Canada	SP in a rheumatoid arthritis patient with severe osteoporosis	[78]
2022	Chile	Nasal carriage of MRSP by veterinarians and dog owners	[79]
2022	Taiwan	Colonization of dog owners by SP	[80]
2022	Nigeria	Nasal carriage of MRSP by dog guardians in dog-owing households	[20]
2022	Netherlands	Colonization of dog owners by SP in dog-owing households	[81]
2022	Germany	*Nasal colonization of humans by S. pseudintermedius in three cohort studies.*	[82]
2022	Canada	Urinary tract infection by SP in a human male patient	[16]
2022	Israel	First case of MRSP in a 12-year-old oncology patient	[13]
2022	USA	A case of SP in a 60-year-old patient with necrotising pneumonia	[83]

MRSP, methicillin-resistant *S. pseudintermedius*; SP, *S. pseudintermedius*; SSTIs, skin and soft tissue infections; and ST, sequence type.

## Data Availability

Not applicable.

## Supplementary Materials

The following supporting information can be downloaded at: https://www.mdpi.com/article/10.3390/microorganisms11030581/s1.
